# Transmigration across a Steady-State Blood–Brain Barrie Induces Activation of Circulating Dendritic Cells Partly Mediated by Actin Cytoskeletal Reorganization

**DOI:** 10.3390/membranes11090700

**Published:** 2021-09-13

**Authors:** Megha Meena, Mats Van Delen, Maxime De Laere, Ann Sterkens, Coloma Costas Romero, Zwi Berneman, Nathalie Cools

**Affiliations:** 1 Laboratory of Experimental Hematology, Vaccine & Infectious Disease Institute (VAXINFECTIO), Faculty of Medicine and Health Sciences, University of Antwerp, 2610 Wilrijk, Belgium; meghameena259@gmail.com (M.M.); Mats.VanDelen@student.uantwerpen.be (M.V.D.); maxime.deLaere@uza.be (M.D.L.); ann.sterkens@uza.be (A.S.); colomacostasromero@gmail.com (C.C.R.); Zwi.Berneman@uza.be (Z.B.); 2 Center for Cell Therapy and Regenerative Medicine, Laboratory of Experimental Hematology, Antwerp University Hospital, 2650 Edegem, Belgium; 3 Department of Dermatology, Antwerp University Hospital, 2650 Edegem, Belgium

**Keywords:** central nervous system, dendritic cells, blood–brain barrier, immune cells, endothelial cells, lymphocytes, transmigration, actin restructuring

## Abstract

The central nervous system (CNS) is considered to be an immunologically unique site, in large part given its extensive protection by the blood–brain barrier (BBB). As our knowledge of the complex interaction between the peripheral immune system and the CNS expands, the mechanisms of immune privilege are being refined. Here, we studied the interaction of dendritic cells (DCs) with the BBB in steady–state conditions and observed that transmigrated DCs display an activated phenotype and stronger T cell-stimulatory capacity as compared to non-migrating DCs. Next, we aimed to gain further insights in the processes underlying activation of DCs following transmigration across the BBB. We investigated the interaction of DCs with endothelial cells as well as the involvement of actin cytoskeletal reorganization. Whereas we were not able to demonstrate that DCs engulf membrane fragments from fluorescently labelled endothelial cells during transmigration across the BBB, we found that blocking actin restructuring of DCs by latrunculin-A significantly impaired in vitro migration of DC across the BBB and subsequent T cell-stimulatory capacity, albeit no effect on migration-induced phenotypic activation could be demonstrated. These observations contribute to the current understanding of the interaction between DCs and the BBB, ultimately leading to the design of targeted therapies capable to inhibit autoimmune inflammation of the CNS.

## 1. Introduction

The blood–brain barrier (BBB) is a highly intricate, active interface between the circulation and the central nervous system (CNS), which restricts the free movement of pathogens, circulating immune cells, and biologically active factors between the bloodstream and the brain microenvironment [1]. In doing so, the BBB plays a crucial role in the maintenance of the (immunological) homeostasis of the CNS, rendering the CNS an immune-privileged and immunologically unique site [2,3]. Whereas the anatomical and functional basis of BBB lies in the tight junctions formed between endothelial cells and their low pinocytotic activity [4], these endothelial cells are in intimate contact with vascular cells (pericytes and vascular smooth muscle cells), glial cells consisting of microglia, astrocytes, and oligodendrocyte lineage cells, and neurons [5,6,7]. These components altogether maintain the structure and integrity of the BBB [8]. The crosstalk and molecular signaling between these different cell types are collectively known as the neurovascular unit, which allows the BBB to properly perform its fundamental physiological functions. Other than that, pericytes play a critical role during angiogenesis and regulating immune cell infiltration [9]. In addition, these cells have been shown to be important for regulating the formation of the BBB during development, as well as maintaining its function in adulthood and aging [10]. Astrocytes, which ensheath almost 90% of brain microvasculature, are part of glial cells and extend many branching cellular processes, including astrocytic end-feet [11,12]. Particularly, astrocytic end-feet establish a close interaction with endothelial cells through transmembrane proteins anchoring, such as the water channel aquaporin-4 and the potassium channel KIR4.1 [13,14]. They also secrete angiopoietin-1 and angiotensin that restrict BBB permeability by supporting efficient organization of tight junctions [15].

To date, it is generally accepted that the CNS is under active surveillance rather than fully immune quiescent [16,17,18]. Indeed, despite the presence of a physical barrier, interactions between the CNS and the peripheral immune system occur, and these are not limited to pathology but also extend to homeostatic functions. In this context, peripheral immune cells cross the BBB and enter the steady-state CNS through mechanisms similar to those seen in peripheral organs, albeit at a lower rate [19]. On the other hand, due to its highly dynamic nature and sensitivity to pro-inflammatory stimuli, the BBB ensures enhanced recruitment of immune cells to resolve local insults that would disrupt homeostasis and optimal functioning of the CNS.

Of particular interest is the migration of dendritic cells (DCs) into and out of the CNS. These cells are involved in both immune-inducing and regulatory responses and are the most potent immune cells in terms of antigen presentation to and activation of T cells. As such, they critically regulate the balance between immunity and tolerance [20,21]. This also explains why these cells play a pivotal role in the immunopathogenesis of several neuroinflammatory disorders, including diseases such as multiple sclerosis (MS) [22,23], Alzheimer’s disease [24], and Parkinson’s disease [25]. In steady-state conditions, DCs are found in low numbers in the meninges, choroid plexus, and cerebrospinal fluid (CSF) [26]. In addition, they appear to migrate to the perivascular compartment, and stay in situ with a *t*_1/2_ of 5–7 days [27,28]. Previously, Zozulya et al. [29] found that the migration of murine in vitro bone marrow-derived DCs across a murine cerebral microvascular endothelial cell monolayer is regulated by CCL3. Importantly, transmigration of DCs upregulated the expression of costimulatory molecules and enhanced their T-cell stimulatory capacity. Nonetheless, the effects of BBB transmigration of DCs circulating in blood from human volunteers remains to be explored.

During the transmigration of leukocytes across the BBB, endothelial cells closely interact with migrating immune cells in various ways. For instance, endothelial cells (ECs) shred microparticles, which are known to affect a variety of immune cells. Indeed, it has been reported that human brain microvascular endothelial cell-derived microparticles could interact with and support the proliferation of T cells. Endothelial cell-derived microparticles can express molecules important for antigen presentation and T cell co-stimulation, such as MHC class II and CD40, and consequently induce T cell activation [30]. Moreover, it is also reported that endothelial cell-derived microparticles can specifically induce plasmacytoid dendritic cell maturation and their production of inflammatory cytokines [31]. In addition, Kedl et al. [32] demonstrated that migratory DCs acquire and present lymphatic endothelial cell-archived antigens during lymph node contraction. Subsequently, migratory DCs cross-present the lymphatic endothelial cell-archived antigens to circulating T cells. These findings prompted us to investigate the influence of the transmigratory process on DCs and to investigate if DCs engulf endothelial fragments from the in vitro BBB endothelial cells while transmigrating, potentially resulting in an altered phenotype and activation status.

Cell migration and cell–cell interactions require dynamic reorganization of the cell’s actin cytoskeleton [33]. Indeed, DC motility relies critically on the actin cytoskeleton, which is, to an important extent, regulated by the actin-related protein 2/3 (ARP2/3) complex, a nucleator of branched actin networks [34,35]. Consequently, loss of ARP2/3 stimulators and upstream Rho family GTPases dramatically impairs DC migration [36]. Besides, studies have shown that the DC actin cytoskeleton and in particular the F-actin network play a role in CD8+ T cell activation by DC and promotes DC-T cell adhesion by constraining ICAM-1 mobility [37,38]. In order to gain a deeper understanding of the process of DC migration and to investigate whether any changes in the DC activation state following transmigration are a direct consequence of actin cytoskeleton restructuring, we studied the effects of latrunculin-A pretreatment preceding migration. Latrunculin-A is an inhibitor of actin polymerization, which disrupts microfilament organization in cultured cells by binding to monomeric G-actin in a 1:1 complex at sub micromolar concentrations [39]. Latrunculin treatment leads to a complete depletion of the F-actin network in the cells and is therefore fit to study the importance of the cytoskeleton remodeling in migration-induced DC activation.

Overall, in this study, we report differences in the activation state of human DCs following migration through a steady-state BBB. Next, we investigated whether this phenomenon can be attributed to the interaction of DCs with endothelial cells while transmigrating and studied the involvement of actin cytoskeleton remodeling. A clear understanding of the alterations caused in DC phenotype and function following their migration may advance the development of new therapies that intervene with the observed transmigration-mediated activation of DCs thereby modulating subsequent potentially autoreactive immune responses.

## 2. Material and Methods

### 2.1. Cell Culture Model of Blood-Brain Barrier (BBB)

**Figure 1:**
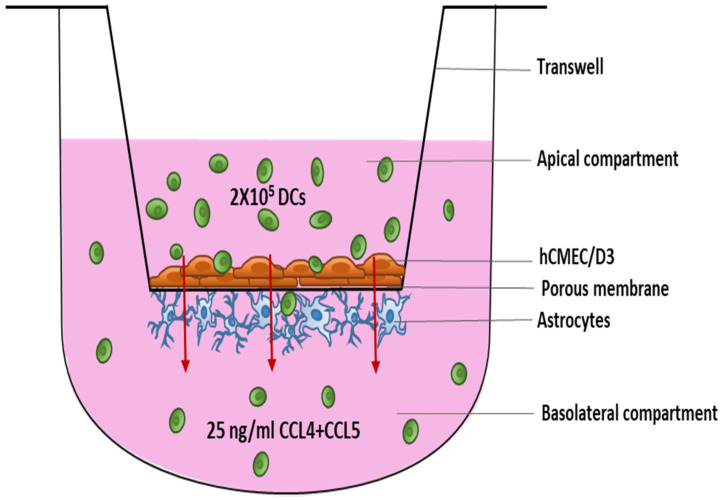
Schematic overview of the DC migration experiment using an in vitro model of the BBB. Endothelial cells were seeded on top of the 3.0 µm porous membrane in a 24-well transwell with astrocytes on the underneath. BBBs were maintained in culture for 10–12 days before the migration of DCs was studied. Abbreviations used: BBB—Blood–brain barrier, DC—Dendritic cells.

The in vitro BBB model was constructed as described previously by our group [40] and others [41]. In brief, human primary astrocytes (Sanbio, Uden, The Netherlands) were seeded at a density of 15,000 cells/cm^2^ on the poly-L-lysine-coated outside of a transwell (24-well format) with 3.0 µm pore size (Greiner Bio-one, Vilvoorde, Belgium) and allowed to adhere for 2 h. Subsequently, transwell inserts were transferred into a well filled with endothelial cell growth medium [EGM]-2-MV medium (Lonza, Verviers, Belgium) with 2.5% fetal bovine serum [FBS] (Thermo Fisher Scientific, Erembodegem, Belgium) and human cerebral microvascular endothelial cell line [hCMEC/D3] (Tébu-bio, Le Perray-en-Yvelines, France) were seeded onto the insert’s collagen-coated inside at a density of 25,000 cells/cm^2^ (Figure 1). Mycoplasma contamination was checked on both the cell lines after they were received using a MycoFluor™ Mycoplasma Detection Kit (Thermo Fisher Scientific, Erembodegem, Belgium).

Cultures were maintained in EGM-2-MV medium in 5% CO_2_ at 37 °C. Three days after initiating the coculture, the growth medium was replaced by endothelial basal medium [EBM-2]-plus, consisting of EBM-2 medium (Lonza) supplemented with 1.4 μM hydrocortisone (Sigma-Aldrich BVBA, Overijse, Belgium), 1 ng/mL basic fibroblast growth factor (bFGF, Thermo Fisher Scientific, Paisley, UK), 10 μg/ml gentamicin, 1 μg/mL amphotericin-B, and 2.5% FBS. EBM-2-plus was replenished every other day.

To check the efficiency of BBB formation, we measured the transendothelial electrical resistance (TEER) at different time points after establishing the BBB coculture. TEER was determined using an EVOM-2 voltohmmeter with STX electrodes (World Precision Instruments, Hitchin, Hertfordshire, UK). Measurements were performed in duplicate and the final mean TEER value is expressed in Ωcm^2^. Background TEER values, i.e., mean TEER across an empty insert, was subtracted from the mean TEER value recorded across BBB cocultures. A constant increase in the measurements was seen on incremental times of culture, which ranged from 10.2 ± 0.78% Ωcm^2^ at day-2 of culture to 29.8 ± 1.05% Ωcm^2^ at day-13 of culture (Supplementary Figure S1). Migration assays were performed between days 10 and 12 of culture, when the cocultures established functional barrier properties as confirmed by stable elevated TEER values (Supplementary Figure S1).

### 2.2. Cell Isolation

Peripheral blood from healthy donors was obtained from buffy coats provided by the Red Cross donor center (Red Cross-Flanders, Mechelen, Belgium), and peripheral blood mononuclear cells (PBMC) were isolated by density gradient centrifugation (Ficoll Pacque PLUS, GE Healthcare, Amsterdam, The Netherlands). The Pan-DC enrichment kit (Miltenyi biotech) was used to isolate DCs from the PBMC. From the remaining PBMC fraction, peripheral blood lymphocytes (PBLs) were depleted from CD14+ monocytes using CD14+ immunomagnetic selection (CD14 Reagent, Miltenyi Biotec, Bergisch Gladbach, Germany), according to the manufacturer’s instructions. The CD14-depleted cell fraction (i.e., peripheral blood lymphocytes (PBLs)) was cryopreserved in FBS supplemented with 10% dimethyl sulfoxide (DMSO, Sigma-Aldrich, Bornem, Belgium) and stored at −80 °C for later use in an allogeneic mixed leukocyte reaction (allo-MLR).

### 2.3. Migration Assay

Transmigration of the isolated DCs was studied across steady-state BBB cocultures. On the day of performing the migration assay, the cocultures were chemokine-coated by the addition of 2.5 ng/mL CCL4 (R&D systems, Bio-techne, Abingdon, UK) and CCL5 (R&D systems, Bio-techne, Abingdon, UK) to the upper transwell compartment for 1 h. Next, BBB cocultures were washed twice with Iscove’s Modified Dulbecco’s Medium (IMDM) (Thermo Fischer Scientific) supplemented with 1% human AB serum (hAB) (Thermo Fischer Scientific) to remove unbound chemokines. These BBB cocultures were then transferred to a new plate where the basolateral compartment contained 25 ng/mL CCL4 and 25 ng/mL CCL5 in IMDM, supplemented with 1% hAB serum. As compared to physiological levels [42,43,44], the used concentrations are relatively high, but the mentioned concentrations are well within the range for which maximal bioactivity was shown for these chemokines by the manufacturer (R&D systems, Bio-techne).

Subsequently, 2 × 10^5^ enriched DCs resuspended in IMDM supplemented with 1% hAB serum were added to the upper compartment. As a negative control, 2 × 10^5^ DCs were added to the upper compartment in the absence of chemokines in the basolateral compartment. As a positive control, 2 × 10^5^ DCs were added directly to the lower compartment. After 20–24 h, migrated cells were collected from the basolateral compartment, while non-migrating cells were recovered from the upper compartment. In addition, DC were tested in a chemotaxis experiment in the absence of the BBB. Harvested cells were counted using a Neubauer counting chamber (Marienfeld, Germany) and the viability of the cells was evaluated using flow cytometric analysis of propidium iodide staining (CytoFLEX, Beckman Coulter, Suarlée, Belgium). The percentage migration was calculated as follows: [(# migrated cells from the experimental sample − # migrated cells from negative control)/# migrated cells from positive control] × 100%.

Where indicated, DCs were pre-treated with 20 μM latrunculin-A (Sigma-Aldrich, Overijse, Belgium) for 1 h at 37 °C and washed three times before adding to the BBBs.

### 2.4. Fluorescent Labeling of Endothelial Cell Layer

The endothelial cell layer in the in vitro BBB was fluorescently labelled, on the day of migration and prior to the addition of DCs to the BBBs, using PKH-67 (Sigma-Aldrich, Overijse, Belgium). For this, 1 µM PKH67 dye was added to the transwells in which endothelial cells formed a confluent cell layer, for 5 min at room temperature, according to the manufacturer’s instructions. The cells were then washed with pure FBS and subsequently EGM-2 + 5% FBS. PKH-67 fluorescence uptake by the endothelial cells in the BBB was checked using an EVOS fluorescence microscope (Thermo Fischer Scientific, Belgium), prior to DC migration across the fluorescently labeled endothelial cell layer of the BBB.

### 2.5. Flow Cytometry

The purity of DCs following pan-DC enrichment was determined using the following fluorochrome-labelled mouse anti-human monoclonal antibodies: Anti-CD303 (BDCA-2)-fluorescein isothiocyanate (FITC) (Miltenyi Biotec, Bergisch Gladbach, Germany), anti-CD1c (BDCA-1)-phycoerythrin (PE) (Miltenyi Biotec, Bergisch Gladbach, Germany), anti-CD141 (BDCA-3)-FITC (Miltenyi Biotec), and anti-human leukocyte antigen (HLA-) DR-peridinin chlorophyll (PerCP, BD Biosciences, Erembodegem, Belgium). Living cells were identified using the 7AAD dye (BD Pharmingen, Erembodegem, Belgium). Next, % positive cells were determined by gating on living cells followed by leukocyte scatter and single-cell gating (Supplementary Figure S2).

The phenotype of DCs was characterized using the following fluorochrome-labelled mouse anti-human monoclonal antibodies: Anti-CD86-FITC (BD Pharmingen, Erembodegem, Belgium), anti-CD80-PE (BD Pharmingen, Erembodegem, Belgium), anti-HLA-DR-PerCP (BD Biosciences, Erembodegem, Belgium), and anti-CD40-PE (BD Pharmingen, Erembodegem, Belgium).

Isotype-matched control monoclonal antibodies were used to determine non-specific background staining. Leukocytes were identified based on forward and side scatter plot of which single cells were gated. These singlets were then further used for the gating the cells of interest based on isotype controls. Propidium iodide (PI) (Invitrogen™, Thermo Fischer) staining was used to check the viability of cells (Supplementary Figure S2).

For analytical flow cytometry, at least 5000 events were analyzed using a flow cytometer (CytoFLEX). All results were analyzed using FlowJo software^TM^ 10.6.2 (Tree Star, Ashland, OR, USA).

To check the engulfment of fluorescently labelled endothelial cells by DCs, the percentage of PKH-67 positive cells was evaluated on a flow cytometer for both the migrated and the non-migrated DC populations. The viability of the two populations of DCs was assessed using flow cytometric analysis of propidium iodide staining on the cells. The fluorescently labelled endothelial cells were harvested from the BBBs following DC migration and further checked for the PKH-67 expression. For this, the astrocytes were removed mechanically from the insert underside and the hCMEC/D3 cells cultured on the upper side of the membrane were homogenized with trypsin-EDTA (Thermo Fischer Scientific). The harvested cells were then washed before flow cytometric analysis. Further, the percentage PKH-67 positive cells were analyzed on the FITC channel against a side scatter plot on the flow cytometer.

### 2.6. Allogeneic Mixed Lymphocyte Reaction

To assess the allogeneic T cell-stimulatory capacity of DCs, DCs were cocultured with allogeneic PBL in a 1:10 ratio. Non-stimulated responder PBL served as a negative control, while allogeneic PBL stimulated with 1 µg/mL phytoheamagglutinin (PHA) (Sigma-Aldrich, Overijse, Belgium) were used as a positive control. Cocultures were performed in IMDM supplemented with 5% hAB serum at 37 °C. After 5 days, the secreted level of IFN-γ in the cell culture supernatant was determined as a measure of T cell stimulatory capacity using a commercially available ELISA kit (PeproTech, East Windsor, NJ, USA).

### 2.7. Statistical Analyses

Data were analyzed using the Graphpad Prism software version 5.01 (Graphpad, San Diego, CA, USA). The normality of data was checked using the Shapiro–Wilk normality test. For the comparison of 2 groups, a paired Student’s *t*-test or a Mann–Whitney U test was used based on the normality of dataset. For comparing 3 groups or more, statistical analysis was performed by one-way ANOVA, followed by Tukey’s multiple comparisons test or by a Kruskal–Wallis test in combination with Dunn’s multiple comparison test in case data were not normally distributed. *p*-value < 0.05 was considered as statistically significant.

## 3. Results

### 3.1. Dendritic Cells That Migrate across the BBB Are in a More Activated State Than Non-Migrating Dendritic Cells

To investigate whether the migration of DCs through the in vitro model of the BBB affects their activation state, we investigated the phenotype and function of BBB-transmigrating DCs as compared to non-migrating DCs. For this, DCs were selectively enriched, resulting in a pure DC population as determined by the expression of specific markers for different DC subsets. This population consisted of 35.4 ± 5.46% of CD1c (BDCA-1)-positive myeloid DCs, 4.8 ± 0.92% of CD141 (BDCA-3)-positive myeloid DCs, and 37.04 ± 2.88% of CD303 (BDCA-2)-positive plasmacytoid DCs, comprising a total of 84.75 ± 5.42% HLA-DR-positive DCs (Supplementary Figure S3). Cells had a viability of 89.23 ± 2.51%.

**Figure 2:**
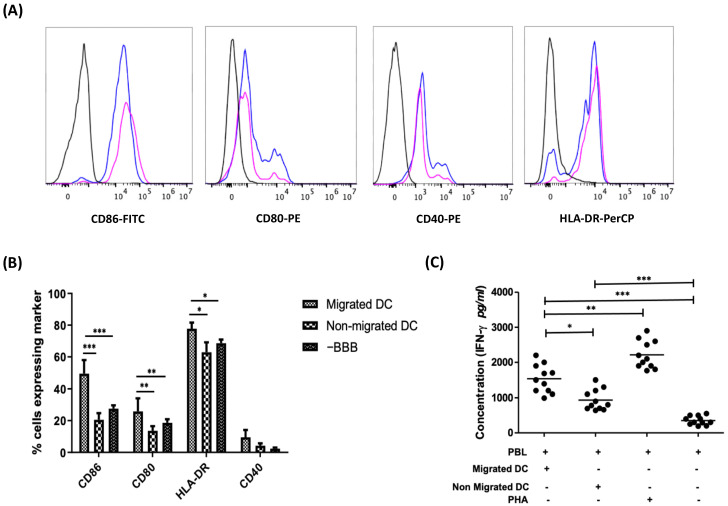
BBB-transmigratory DCs display a more activated state as compared to their non-migrating counterparts and when compared to the migrating DCs in the absence of BBBs. (**A**) Representative figure showing the expression of co-stimulatory markers of migrating DCs (blue peak) and non-migrating dendritic cells (pink peak) as compared to their respective isotype controls (black). The count numbers of these cells are provided in Table S1 of the supplementary information (Table S1). (**B**) The expression of the activation markers CD80, CD86, HLA-DR, and CD40 on migrating vs. non-migrating DCs following a chemotaxis assay in an in vitro BBB model was evaluated along with the comparison against a control sample in the absence of BBB. Migrated DCs demonstrate a significantly higher expression of CD80, CD86, and HLA-DR as compared to non-migrated DCs (*n* = 6) and the DCs migrating in the absence of BBB (*n* = 3). (**C**) DCs that migrate across an in vitro model of the BBB demonstrate stronger T cell-stimulatory capacity as compared to non-migrating DCs, as shown in an allo-MLR (*n* = 11). (* *p* < 0.05; ** *p* < 0.01; *** *p* < 0.001).

Following chemokine-stimulated transmigration across the in vitro steady-state BBB and in the absence of BBB, non-migrating and migrating DCs were collected for flow cytometric analysis. On average, 14.55 ± 3.25% of DCs migrated across the in vitro BBB. No significant difference was seen between the viability of the non-migrating (76.89 ± 3.92%) and the migrating DC populations (78.6 ± 3.67%). Interestingly, the expression of CD80 (*p* < 0.01), CD86 (*p* < 0.001), and HLA-DR (*p* < 0.05) was significantly upregulated on migrating DCs as compared to the non-migrating DCs as well as to DC migrating in the absence of BBB-composing cells, albeit no statistically significant difference in CD40 expression was observed (Figure 2B). Moreover, no significant difference in the expression level of the markers tested was found when DCs were treated with CCL4 and CCL5 directly as compared to non-chemokine treated DCs (data not shown). Altogether, our findings suggest that DCs have a more activated phenotype after passing through the BBB.

Next, we investigated if DCs that migrate through the BBB are also functionally more active. For this, we compared the T cell-stimulatory capacity of migrating DCs with the non-migrating DC population. IFN-γ secretion by allogeneic PBLs stimulated with DCs that migrated across the BBBs as compared to those stimulated with non-migrating DC was used as a measure for T cell-stimulatory capacity of DCs. We observed significantly higher levels of IFN-γ secreted by responder PBLs stimulated with the migrating DC population as compared to PBLs stimulated with non-migrating DCs (*p* < 0.05; Figure 2C), indicative of a stronger T cell-stimulatory capacity of DCs that migrated across the BBB.

### 3.2. Dendritic Cells Do Not Take up Membrane Fragments of Endothelial Cells following Transmigration

**Figure 3:**
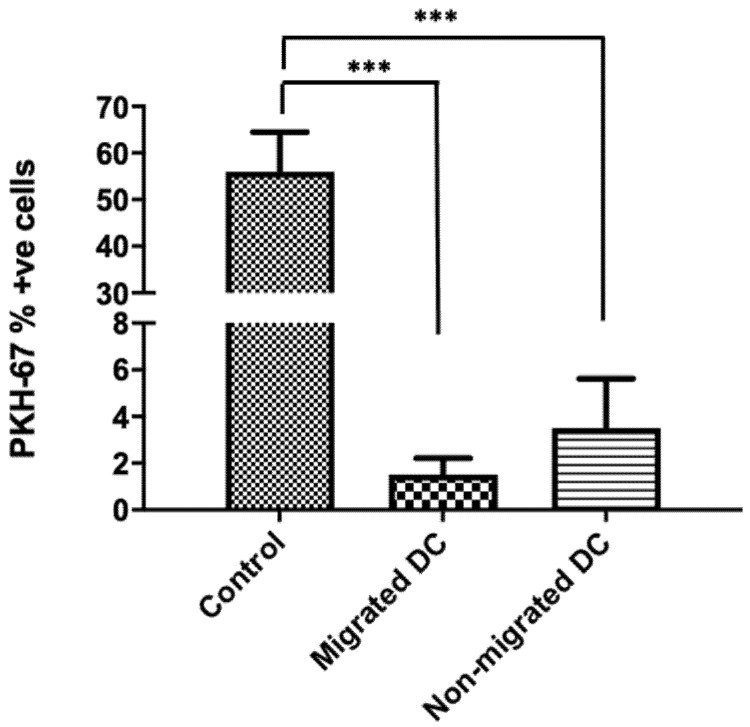
DCs do not engulf endothelial cells while transmigrating across a steady-state BBB. Migrated and non-migrated DC were harvested after a 22-h migration assay across an intact in vitro BBB (*n* = 7). The expression of PKH-67 was assessed on the collected migrated and non-migrated DC populations. Only a minority of DCs were PKH-67 positive. Control samples were from the fluorescently labelled endothelial cells harvested from the BBB. (*** *p* < 0.001).

Next, we studied if the migrating DCs capture membrane fragments from the endothelial cells while moving across the BBB and whether this contributed to the activation of the DCs. For this, we fluorescently labelled the endothelial cells in the in vitro transwell model of the BBB with the cell membrane labelling dye PKH-67 in order to check the interactions between endothelial cells and DCs. Membrane staining of the endothelial cell layer was confirmed using an EVOS fluorescence microscope prior to migration (Supplementary Figure S4), as indicated by 56.74 ± 2.57% of endothelial cells that are fluorescently labeled. Next, the proportion of DCs exhibiting fluorescence following migration across the brain microvessel endothelium was assessed. We observed that very few of the migrated DCs (1.89 ± 1.73%) and non-migrated DCs (5.5 ± 3.31) exhibited membrane fluorescence from the dye (Figure 3). The viability of the migrated DCs (78.5 ± 2.33%) was similar to the non-migrated DCs (75.31 ± 1.8%) across the fluorescently labelled BBBs. This suggests that DCs that transmigrate across the BBBs do not engulf membrane components from endothelial cells at a significant rate.

### 3.3. Actin Cytoskeleton Restructuring of DCs Has No Effect on Migration-Induced Phenotypic Activation but Governs DC Migration and T Cell-Stimulatory Capacity

**Figure 4:**
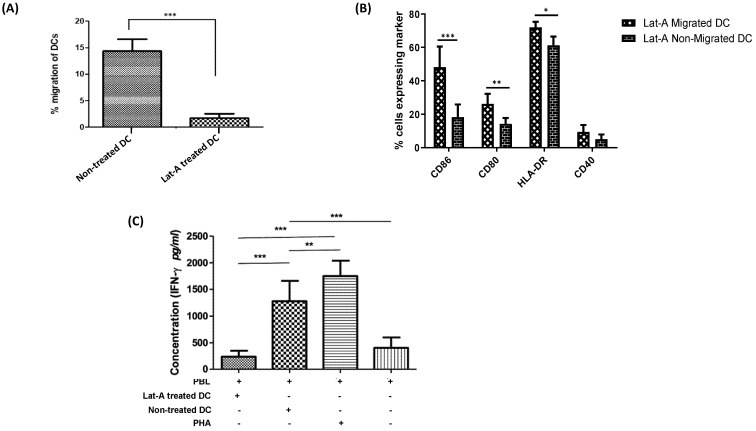
Actin cytoskeleton restructuring of DCs has no effect on migration-induced phenotypic activation but governs DC migration and T cell-stimulatory capacity. (**A**) Pre-treatment of DCs with latrunculin-A significantly reduces migratory capacity (*n* = 5). (**B**) Following a chemokine-driven assay in an in vitro BBB model, a comparison was made against the migratory and non-migratory DCs treated with latrunculin-A. A significantly higher phenotypic expression of activation markers CD86, CD80 along with HLA-DR was observed in the migratory DCs when compared to the non-migratory DCs with latrunculin-A pre-treatment (*n* = 5). (**C**) In an allo-MLR, responder PBLs stimulated with latruncilin-A treated DCs secreted significantly lower levels of IFN-γ as compared to PBLs stimulated with untreated DCs. Allogeneic PBLs stimulated with 1 µg/mL of phytoheamagglutinin served as a positive control and the non-stimulated responder PBLs were used as a negative control (*n* = 4). (* *p* < 0.05; ** *p* < 0.01; *** *p* < 0.001).

For better understanding of the influence of DC cytoskeleton reorganization on DC activation and maturation along with the transmigratory capacities of DCs, we treated DCs with a cytoskeleton inhibitor latruncilin-A prior to their migration across the in vitro BBB. It was observed that the migration of DCs was severely disrupted upon treatment with latruncilin-A when compared to non-treated DCs (Figure 4A). The migratory capacity of latruncilin-A treated DCs (2.53 ± 0.77%) was significantly lower (*p* < 0.001) than that of untreated DCs (13.89 ± 4.17%). Besides, the viability of the migrated DC population treated with latruncilin-A (68.19 ± 1.82%) was similar to the viability of non-migrating latruncilin-A treated DCs (74.89 ± 3.16%) and to the non-treated cells (77.32 ± 4.22%). Additionally, DCs that were treated with latruncilin-A exhibited no significant differences in the expression levels of the co-stimulatory markers, CD80, CD86, HLA-DR, and CD40 as compared to non-treated DCs for both the migrated and non-migrated DC subsets. Nonetheless, the initially observed phenotypic differences between migrating and non-migrating DCs persisted after latruncilin-A treatment (Figure 4B). To test the effect of latruncilin-A on the T-cell stimulatory capacity of DCs, we performed an allo-MLR of PBLs stimulated with latruncilin-A treated and untreated DCs. We observed that the level of secreted IFN-γ in the supernatant of PBLs stimulated with latruncilin-A treated DCs was significantly lower (*p* < 0.001) as compared with that of PBLs stimulated with untreated DCs (Figure 4C). This suggests that along with a hindered migratory capacity of DCs, latruncilin-A treatment also majorly interrupts the T-cell stimulatory capacity of DCs and henceforth are functionally inactive, despite an unaffected phenotypic activation status.

## 4. Discussion

The CNS was once considered to be an area of absolute immune privilege, where the endothelial, epithelial, and glial brain barriers strictly prevented immune cell entry into the different compartments of the CNS. Despite the presence of physical barriers in the CNS, a low number antigen-presenting DCs are found in perivascular spaces serving as sentinels guarding the CNS in the steady state. The extravasation of DCs, as for any type of circulating immune cell, is a multi-step process that has extensively been described [5,45], but the consequences of DC migration across the BBB at the DC level are inadequately understood. A coherent understanding is crucial for gaining increased insight in the lifecycle of DCs in the CNS during homeostasis and pathology, and for the development of therapeutic strategies, which can correct any imbalances occurring during CNS inflammatory disorders. To procure a better perception of the process of DC transmigration across the BBB in steady-state conditions, we used an in vitro model of the BBB, consisting of a coculture of the cerebral microvascular endothelial cell line hCMEC/D3 and primary human astrocytes. In doing so, we studied the phenotypic and functional differences caused in the profile of circulating DCs following their migration into the CNS.

Interestingly, we witnessed highly increased expression of the maturation markers CD80, CD86, and HLA-DR on DCs following their migration as compared to the other subset of DCs unable to migrate. Moreover, the migrated DC population showed an elevated functional activation as evidenced by an increased T cell-stimulatory capacity. Although we did not see an effect of migration on the expression of CD40 by DCs, others have demonstrated that CD40 plays a role in the migration process of CD4 T cells across brain microvascular endothelial cells and increases adhesion of resting and activated CD4+ T lymphocytes to endothelium [46]. Similar results were observed by Zozulya and colleagues [29], who used an in vitro model of murine cerebral microvascular endothelial cell monolayers to show that CCL3 can stimulate the transmigration of mature bone marrow-derived DCs in an MMP-dependent manner and equally witnessed DC activation upon transmigration.

Altogether, these results can plausibly demonstrate that DCs acquire a phenotypic and functionally active status post migration across a steady-state BBB in vitro. While immunohistological studies investigating the maturation state of DCs in the CNS in situ have been conflicting [47,48,49,50], a study by Anandasabapathy et al. [27] in mice confirms that in the steady-state CNS, DCs attain a mature phenotype and are capable of stimulating T cells. While in steady-state conditions, this is not associated with autoimmune responses, this finding is of relevance in the context of CNS autoimmune disorders.

Based on previous reports, endothelial cells exchange antigens with DCs and shed microparticles, which lead to plasmacytoid DC maturation [30,31]. In line with this, we hypothesized that endothelial cells could directly lead to the maturation and activation of DCs by imparting their membrane particles to DCs during their migrating across the BBB. We observed that circulating DCs do not engulf any significant amount of membrane fragments from the steady-state endothelial cells during their transmigratory process.

This is opposed to what has been reported previously [31,32]. For instance, Kedl and co-workers [32] demonstrated antigen exchange of viral antigens present on the lymphatic endothelial cell surface, following viral infection in mice, and DCs, and subsequent cross presentation of these antigens by the DCs. Furthermore, others demonstrated upregulated expression of co-stimulatory markers, increased secretion of inflammatory cytokines, and enhanced ability to stimulate naive CD4 T-cell proliferation by plasmacytoid dendritic cells (pDC) following interaction with endothelial cells [31]. For this, microparticles, which are complex vesicular structures shed by endothelial cells, are isolated after activating the endothelial cells, and incubated with human pDC. Subsequently, the uptake of these microparticles by pDC was observed. In contrast, in current study, endothelial cells were not activated, and engulfment of membrane fragments from steady-state endothelial cells directly by the DCs when moving across the BBB was investigated. This could indicate that transmigration of cells, and DCs in particular, occurs via different mechanisms in the steady state versus in an inflammatory environment when endothelial cells are activated. In addition, dendritic cells may not directly engulf the membrane fragments from the endothelial cell layer but are activated by microparticles shed following the activation of the BBB. Hence, very few microparticles might be present in the steady culture of BBB as a result of decreased membrane vesiculation of endothelial cells. Alternatively, fluorescent labelling of endothelial cells might not be sufficient for demonstrating the engulfment of cells or cellular particles by DCs since DCs might have internalized the fragments and presented on their membrane. Consequently, investigating the expression of endothelial cell-specific molecules such as endothelial cell-derived antigens, chemerins, and markers like CD31 and CD99 on the transmigrating DCs could be valuable analysis warranting further investigation of the exact mechanism driving DC activation following transmigration across a steady-state BBB.

Likely, endothelial cells induce DC activation via other mechanisms, which might require direct cell–cell contact or could occur through the secretion of cytokines and signaling molecules by endothelial cells. These mechanisms were not investigated here and still need to be explored. In favor of this hypothesis are earlier studies, which described that a combination of fibroblast, endothelial, and epithelial cell-conditioned media can promote DC maturation when added to the DC culture medium [51] and that the vascular endothelial growth inhibitor (VEGI), which is an anti-angiogenic cytokine produced by endothelial cells, can mediate DC maturation [52]. Other than this, endothelial cells can promote attraction and subsequent maturation of DCs by several other factors in steady-state and inflammatory conditions [53,54,55,56]. Moreover, there could also be the effects of astrocytes from the BBB, which could lead to the activation of DCs following migration, as once DCs cross the blood vasculature, the first cellular structure they encounter are the endfeet or processes of astrocytes. Interestingly, it has been previously reported that human CNS astrocytes could lead to the activation of B cells [57]. Furthermore, astrocytes have been implicated in playing a vital role in antigen presentation and naive T-cell activation [58]. Hence, a direct role of astrocytes in the process of DC maturation and activation following transmigration through a BBB in steady-state and inflammatory conditions cannot be excluded and still needs to be inspected.

The modulation of actin architecture is an essential and fundamental feature of both DC migration and maturation. A previous report by Burns et al. [59,60] showed that changes in the DC actin cytoskeleton facilitate the transition from highly endocytic tissue-resident cells to migratory cells specialized for antigen presentation. This process involves changes in the activation status of Rho GTPases and downstream actin regulatory proteins, and is known to downregulate antigen uptake and increase cell motility [61,62]. Similarly, others previously reported the importance of the actin cytoskeleton in lymphocyte activation [63]. In particular, actin and microtubule meshwork are known to polarize and activate T cells [64,65]. Additionally, the actin cytoskeleton is also known to play a role in the regulation of B cell activation [66].

To investigate if changes in actin cytoarchitecture also result in increased maturation and activation of migratory DCs across a BBB, we treated immature circulating DCs with an actin-depolymerizing agent and a potent cytoskeleton inhibitor, latrunculin-A. The drug did not affect the phenotypic activation of the migratory DCs as compared to the untreated subsets of cells, indicating that the increased phenotypic activation of DCs post migration via the BBB depends on factors other than the actin cytoskeleton restructuring of the cells. In contrast, the drug induced a significant decrease in the T cell-stimulatory capacity of treated DCs compared to the untreated DCs, along with highly disrupted migratory capacity. The significantly lowered T cell stimulation by the latrunculin-A treated DCs could be an effect of the loss of cortical stiffness by the DCs upon treatment. This is based on the findings from Blumenthal et al. [67], which showed that T cell priming is enhanced by maturation-dependent stiffening of the DC cortex. These findings can be used in the future to further unravel the effects of the cytoskeleton formation of DCs and its role in their activation and maturation.

The current study also has some limitations. First, pericytes were not included in the in vitro model of the BBB used here. Nonetheless, previous reports have established that pericytes play a critical role in the integration of endothelial and astrocyte function at the neurovascular unit, and in the regulation of the BBB in vitro [9,11,24]. Hence, including pericytes in an in vitro model of the BBB could provide additional phenotypic advantages in mimicking the in vivo BBB. Second, we incorporated a static in vitro model of the BBB in the current study, lacking unidirectional flow. Indeed, culturing the cells composing the BBB under continuous flow generates shear stress and regulates the expression of transporters and tight junctions, contributing towards effective barrier function. Recently, there have been some breakthrough studies in the development of a 3D organ-on-a-chip model of BBB [68] with a hollow channel in which a continuous monolayer of cells can be grown at the interphase between the lumen and the underlying endothelial cell matrix. Likewise, Wevers and colleagues [69] developed an in vitro model of the human BBB in a high-throughput microfluidic platform. This system is free of artificial membranes, accommodates fluid flow through the blood vessels, and allows fluid-phase sampling of molecules that penetrate the endothelial and matrix layers. Besides this, there have been other recent advancements in human brain organoid models and their application in modelling neurodevelopmental and neurodegenerative diseases [70,71,72]. Importantly, vascularized organoids, which more precisely mimic in vivo brain anatomy and physiology, may facilitate brain disease modelling. Ultimately, these models will lead to the development of efficient high-throughput screening and computer-aided drug design methods [73,74]. The recent advances in these novel methods have the potential to investigate hundreds of thousands of compounds per day hence reducing the cost, time, and effort required to develop new drugs. These systems comprise several steps including target recognition, compound management, reagent preparation, assay development, as well as the screening itself for designing novel therapies for neurodegenerative diseases [74]. This could be an important breakthrough driving the drug discovery arena for neurodegenerative diseases and can provide novel insights in the underlying disease-associated mechanisms supporting the design of targeted therapies in the future.

In conclusion, in this study, we have demonstrated that transmigration of DCs across an in vitro transwell model of the BBB results in the upregulation of the expression of costimulatory molecules and T cell-stimulatory capacity. Other than this, we showed that endothelial cells do not impart their membrane particles to DCs that move across the BBB. Finally, we established that latrunculin-A severely disrupts the migration of DCs across the BBB as well as the T cell-stimulatory capacity of DCs, while not affecting phenotypic maturation as a consequence of transmigration. Understanding the underlying mechanism of the interaction between brain microvessel endothelial cells and DCs might lead to the design of targeted therapies to modify transmigration of DCs in the CNS and thus to inhibit perpetuating autoimmune inflammation of the CNS.

## Data Availability

Not applicable.

## Supplementary Materials

The following are available online at https://www.mdpi.com/article/10.3390/membranes11090700/s1, Figure S1: The transendothelial electrical resistance (TEER) was recorded for the in vitro BBBs from day-2 of coculture. An incremental increase in the TEER values was seen over time. The last point of measurement was at day-12 and day-13 at which a significantly higher TEER value was observed as compared to day-2. This indicates a sufficiently confluent BBB formation, Figure S2. (A) Gating strategy for determination of purity of dendritic cells enriched using the Pan-DC enrichment kit. The cells were stained with 7AAD dye and a clear population of viable cells was observed and gated for further analysis. A forward and side scatter plot was generated from the viable cell population and leukocytes were gated for further analysis. A single cell population was additionally gated from the leukocytes which was then used for gating the % positive cells for their respective fluorophores based on the isotype controls. (B) Gating strategy for characterization of dendritic cells following a migration assay. Leukocytes were identified based on forward and side scatter plot of which single cells were gated. These singlets were then further used for the gating the cells of interest based on isotype controls. In these cells, the viability was additionally checked using PI staining, Figure S3. Representative example of the different isolated circulating DC populations following magnetic Pan-DC enrichment as determined by flow cytometry. The cells were fluorescently stained with anti-CD303-FITC, anti-CD303-PE (BDCA-2), anti-CD1c-PE (BDCA-1), anti-CD141-FITC (BDCA-3) and anti-HLA-DR-FITC, Figure S4. Fluorescent image of endothelial cells labelled with the PKH-67 dye as taken from an EVOS fluorescent microscope, Table S1. Number of events measured to acquire results depicted in Figure 2A in the manuscript ± error on the measurement, according to Poisson distribution of flow cytometry data.
