# Supplementary material for: Transmigration across a Steady-State Blood–Brain Barrie Induces Activation of Circulating Dendritic Cells Partly Mediated by Actin Cytoskeletal Reorganization
Source: Membranes (Basel). 2021 Sep 13;11(9):700. doi: 10.3390/membranes11090700 (PMC8472465; doi:10.3390/membranes11090700)
Supplement: Supplementary file 1 [file membranes-11-00700-s001.zip › membranes-1266224-supplementary.pdf]

membranes

Supplementary File

Transmigration Across a Steady-State Blood-Brain Barrier Induces Activation of Circulating Dendritic Cells Partly Mediated by Actin Cytoskeletal Reorganization

Megha Meena [1], Mats Van Delen [1], Maxime De Laere [1,2], Ann Sterkens [1,3], Coloma Costas Romero [1], Zwi Berneman [1,2] and Nathalie Cools [1,2,*]

[1] Laboratory of Experimental Hematology, Vaccine & Infectious Disease Institute (VAXINFECTIO), Faculty of Medicine and Health Sciences, University of Antwerp, 2610 Wilrijk, Belgium; meghameena259@gmail.com (M.M.); Mats.VanDelen@student.uantwerpen.be (M.V.D.); maxime.deLaere@uza.be (M.D.L.); ann.sterkens@uza.be (A.S.); colomacostasromero@gmail.com (C.C.R.); Zwi.Berneman@uza.be (Z.B.)
[2] Center for Cell Therapy and Regenerative Medicine, Laboratory of Experimental Hematology, Antwerp University Hospital, 2650 Edegem, Belgium
[3] Department of Dermatology, Antwerp University Hospital, 2650 Edegem, Belgium
* Correspondence: nathalie.cools@uza.be

Citation: Meena, M.; Van Delen, M.; De Laere, M.; Sterkens, A.; Costas Romero, C.; Berneman, Z.; Cools, N. Transmigration across a Steady-State Blood-Brain Barrier Induces Activation of Circulating Dendritic Cells Partly Mediated by Actin Cytoskeletal Reorganization. *Membranes* **2021**, *11*, 700. https://doi.org/10.3390/membranes11090700

Academic Editor: Marina Pinheiro and Sandra Silva

Received: 2 June 2021
Accepted: 6 September 2021
Published: 13 September 2021

Publisher's Note: MDPI stays neutral with regard to jurisdictional claims in published maps and institutional affiliations.

Copyright: © 2021 by the authors. Licensee MDPI, Basel, Switzerland. This article is an open access article distributed under the terms and conditions of the Creative Commons Attribution (CC BY) license (https://creativecommons.org/licenses/by/4.0/).

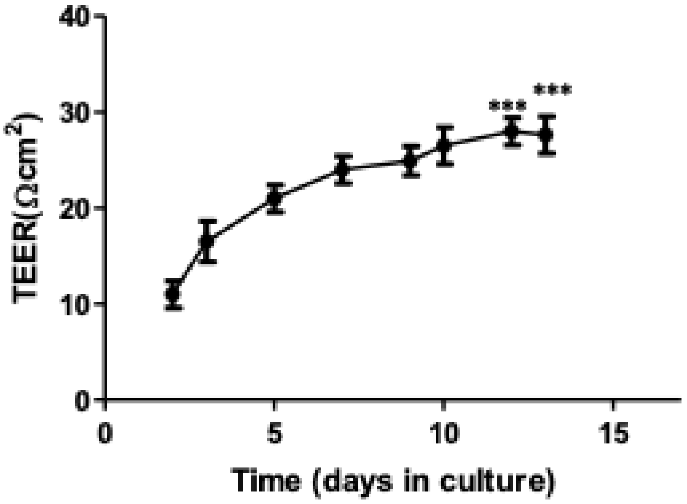

Figure S1. The transendothelial electrical resistance (TEER) was recorded for the in vitro BBBs from day-2 of coculture. An incremental increase in the TEER values was seen over time. The last point of measurement was at day-12 and day-13 at which a significantly higher TEER value was observed as compared to day-2. This indicates a sufficiently confluent BBB formation. (*** $p < 0.001$).

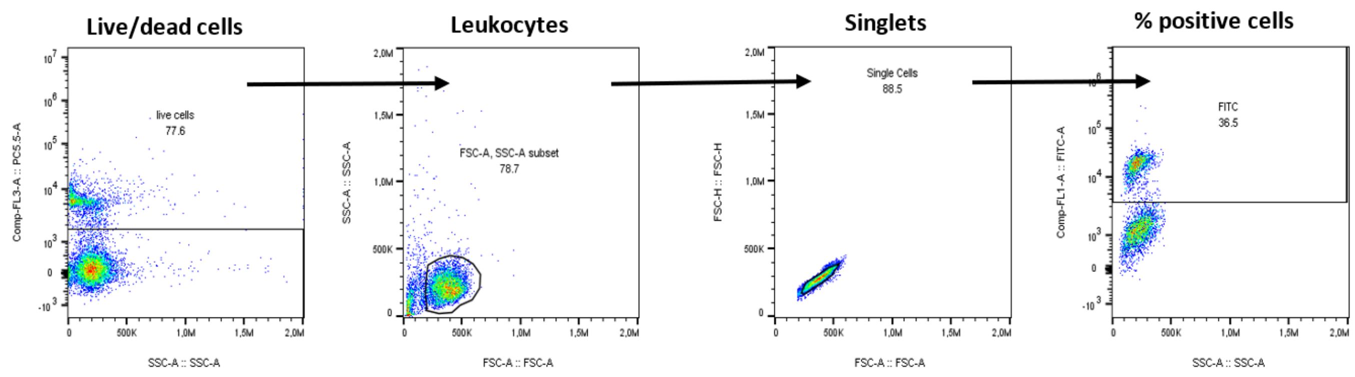

(**A**)

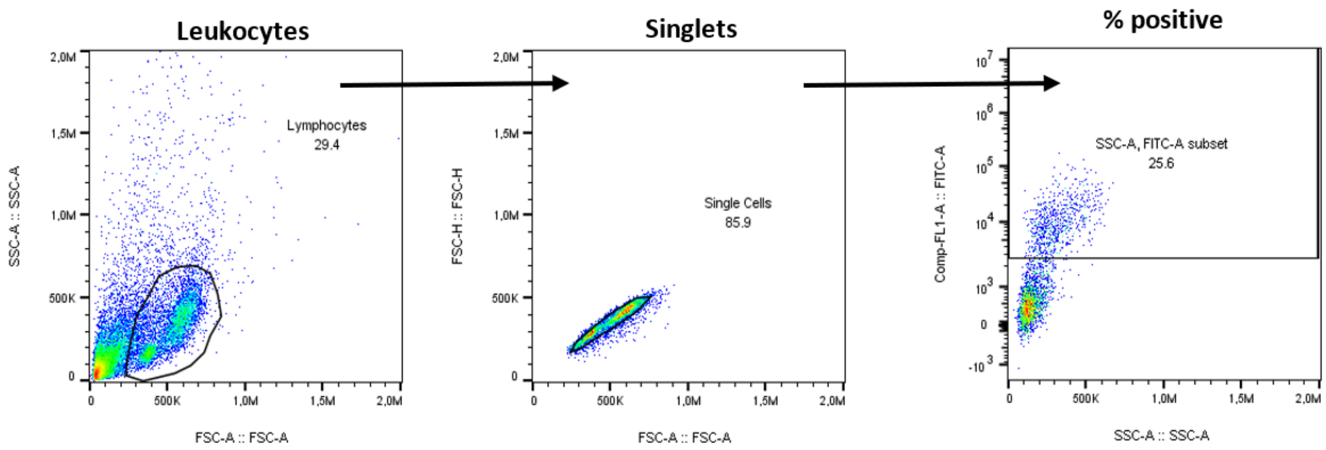

(**B**)

Figure S2. **(A)** Gating strategy for determination of purity of dendritic cells enriched using the Pan-DC enrichment kit. The cells were stained with 7AAD dye and a clear population of viable cells was observed and gated for further analysis. A forward and side scatter plot was generated from the viable cell population and leukocytes were gated for further analysis. A single cell population was additionally gated from the leukocytes which was then used for gating the % positive cells for their respective fluorophores based on the isotype controls. (**B**) Gating strategy for characterization of dendritic cells following a migration assay. Leukocytes were identified based on forward and side scatter plot of which single cells were gated. These singlets were then further used for the gating the cells of interest based on isotype controls. In these cells, the viability was additionally checked using PI staining.

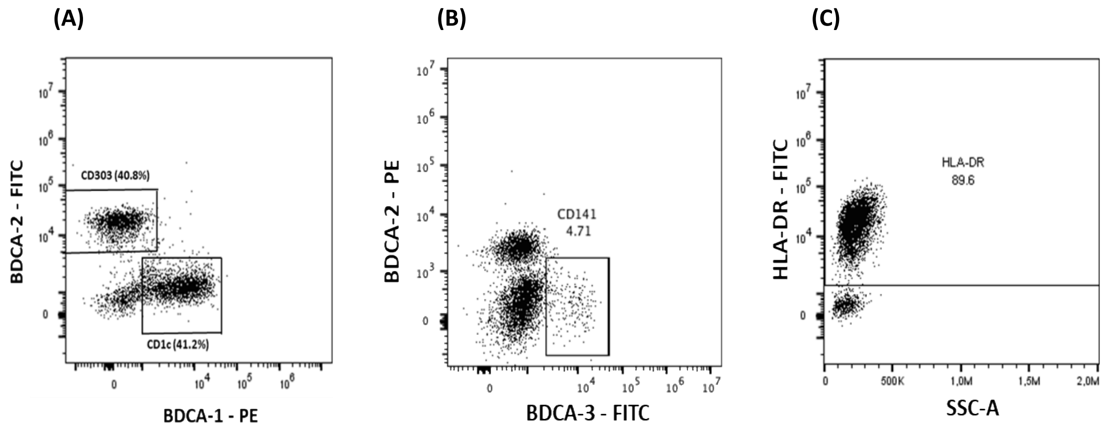

Figure S3. Representative example of the different isolated circulating DC populations following magnetic Pan-DC enrichment as determined by flow cytometry. The cells were fluorescently stained with **(A)** anti-CD303-FITC (BDCA-2) and anti-CD1c-PE (BDCA-1) **(B)** anti-CD303-PE (BDCA-2) and anti-CD141-FITC (BDCA-3) and **(C)** anti-HLA-DR-FITC.

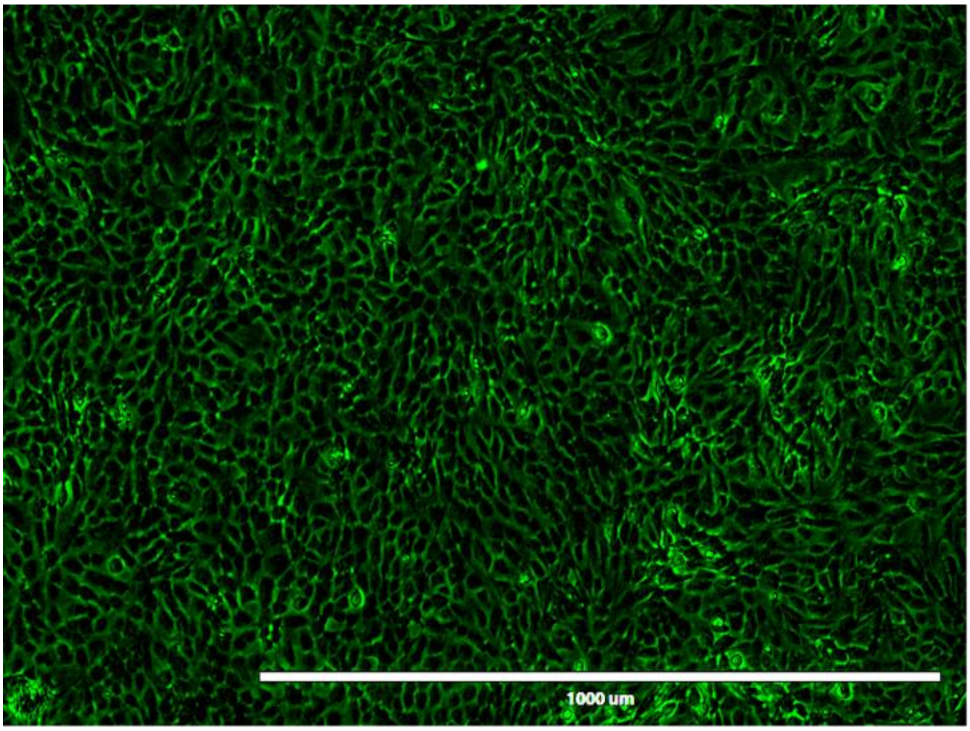

Figure S4. Fluorescent image of endothelial cells labelled with the PKH-67 dye as taken from an EVOS fluorescent microscope.

Table S1. Number of events measured to acquire results depicted in Figure 2A in the manuscript ± error on the measurement, according to Poisson distribution of flow cytometry data.

Conditions	No. of events	Error on the measured events
CD86 labelled Migrated DCs	3850	± 62.0
CD86 labelled Non-migrated DCs	1250	± 35.4
CD80 labelled Migrated DCs	2186	± 46.8
CD80 labelled Non-migrated DCs	778	± 27.9
CD40 labelled Migrated DCs	453	± 21.3
CD40 labelled Non-migrated DCs	327	± 18.1
HLA-DR labelled Migrated DCs	8250	± 90.8
HLA-DR labelled Non-migrated DCs	5978	± 77.3
